# Developmental Plasticity in Response to Embryo Cryopreservation: The Importance of the Vitrification Device in Rabbits

**DOI:** 10.3390/ani10050804

**Published:** 2020-05-06

**Authors:** Ximo Garcia-Dominguez, José Salvador Vicente, Francisco Marco-Jiménez

**Affiliations:** Instituto de Ciencia y Tecnología Animal, Universitat Politècnica de València, 46022 Valencia, Spain; ximo.garciadominguez@gmail.com (X.G.-D.); jvicent@dca.upv.es (J.S.V.)

**Keywords:** assisted reproduction technology, perinatal outcomes, postnatal outcomes, embryo transfer, embryo vitrification

## Abstract

**Simple Summary:**

This study was conducted to demonstrate how embryo manipulation techniques incur phenotypic changes throughout life. This study reports the first evidence demonstrating that the vitrification device used is not a trivial decision, providing valuable information about how the cooling–warming rates during vitrification can be partly responsible of the postnatal phenotypic variations.

**Abstract:**

In this study, we evaluated the effect of embryo vitrification using two different devices on adulthood phenotype in rabbits. In vitro development, prenatal embryo survival, body weight, growth performance, haematological and biochemical peripheral blood analysis, reproductive performance, and lactation performance traits were compared between the experimental groups. They derived from naturally-conceived embryos (NC), fresh-transferred embryos (FT), vitrified-transferred embryos using mini-straw (VTs), or vitrified-transferred embryos using Cryotop (VTc). Straw-vitrified embryos exhibited lower in vitro developmental rates and in vivo survival rates following embryo transfer compared to its Cryotop-vitrified counterparts. Moreover, the VTs group exhibited higher foetal losses than VTc, FT, and NC groups. Independently of the vitrification device, vitrified-transferred (VT) offspring showed a skewed sex ratio in favour of males, and an increased birth bodyweight. In contrast, postnatal daily growth was diminished in all ART (i.e., FT and VT) animals. In adulthood, significant differences in body weight between all groups was founded—all ART progenies weighed less than NC animals and, within ART, VT animals weighed less than FT. For VT groups, weight at adulthood was higher for the VTs group compared with the VTc group. Peripheral blood parameters ranged between common values. Moreover, no differences were found in the fertility rates between experimental groups. Furthermore, similar pregnancy rates, litter sizes, and the number of liveborns were observed, regardless of the experimental group. However, decreased milk yield occurred for VTc and FT animals compared to VTs and NC animals. A similar trend was observed for the milk composition of dry matter and fat. Concordantly, reduced body weight was found for suckling kits in the VTc and FT groups compared to VTs and NC animals. Our findings reveal that developmental changes after the embryo vitrification procedure could be associated with an exhibition of the embryonic developmental plasticity. Moreover, to our best knowledge, this study reports the first evidence demonstrating that the vitrification device used is not a trivial decision, providing valuable information about how the cooling–warming rates during vitrification can be partly responsible of the postnatal phenotypic variations.

## 1. Introduction

Despite advances in assisted reproductive technologies (ART), *in vitro* conditions fail to mimic the optimal physiological dynamism within the reproductive tract [1,2]. In this sense, embryonic plasticity allows embryos to develop responses to ensure their short-term survival in sub-optimal environments [3], which could increase the risk of developmental deviations and disease later in life [4]. Therefore, although ART progenies seem healthy, there is increasing awareness of potential long-term consequences of ART, raising the importance of discerning whether ART leaves a subtle legacy in ART offspring [5].

In humans, it is difficult to determine associations between treatment and outcome, as lifestyle or demographic and clinical factors such as patient infertility can act as potential confounders that bias results [6,7]. Thus, controversially, ART has been linked with adverse obstetric and perinatal outcomes, as well as increased risk of congenital disabilities, cancers, and growth and development disorders [7]. Furthermore, emerging evidence suggests that ART may also predispose individuals to an increased risk of chronic ageing-related diseases such as obesity, type 2 diabetes, and cardiovascular disease. However, recently, the largest studies assessing ART consequences found no alarming evidence in the long-term health outcomes of adults [8,9]. Using fertile and healthy animal models, confounding factors were avoided, thus providing adequate experimental groups to reveal the effects of ART *per se*. Hence, these studies demonstrated both individual and cumulative effects of each ART procedure on foetal and postnatal phenotypes [10,11,12]. 

According to the last report from the European Society of Human Reproduction and Embryology (ESHRE), the steepest increase in treatment numbers was observed in cryopreserved embryo transfer (+13.6%), placing this technique as the second most commonly used in fertility treatments [13]. Unlike most ART trying to mimic the physiological conditions, cryopreservation requires embryo exposure to non-physiologic low temperatures and toxic cryoprotectant solutions to avoid ice-induced injuries [14]. In this context, progenies born after embryo cryopreservation could have an increased risk for many worrisome diseases in comparison to other ARTs [15,16]. In recent years, many laboratories worldwide have completely replaced slow freezing by vitrification because of its improved cryosurvival outcomes [17]. The breakthrough in the field of vitrification came when sample volume was reduced to a level that permitted lowering of the cryoprotectant concentration by maximising cooling and warming rates [18,19]. For this purpose, numerous devices have been described in the literature that minimise the volume of the vitrification solution to allow better heat transfer [18,19]. However, despite all of this effort to reduce embryo damage and increase their survival, consistent long-term follow-up data on the resultant offspring are non-existent. Here, we developed an experimental model approach to evaluate the effects of the embryo cryopreservation procedure, including two clinical vitrification devices, in relation to adulthood phenotype in rabbits.

## 2. Materials and Methods

All the experimental procedures used in this study were performed following Directive 2010/63/EU EEC for animal experiments and were reviewed and approved by the Ethical Committee for Experimentation with Animals of the Universitat Politècnica de Valéncia, Spain (research code: 2015/VSC/PEA/00061).

### 2.1. Experimental Design

**Figure 1:**
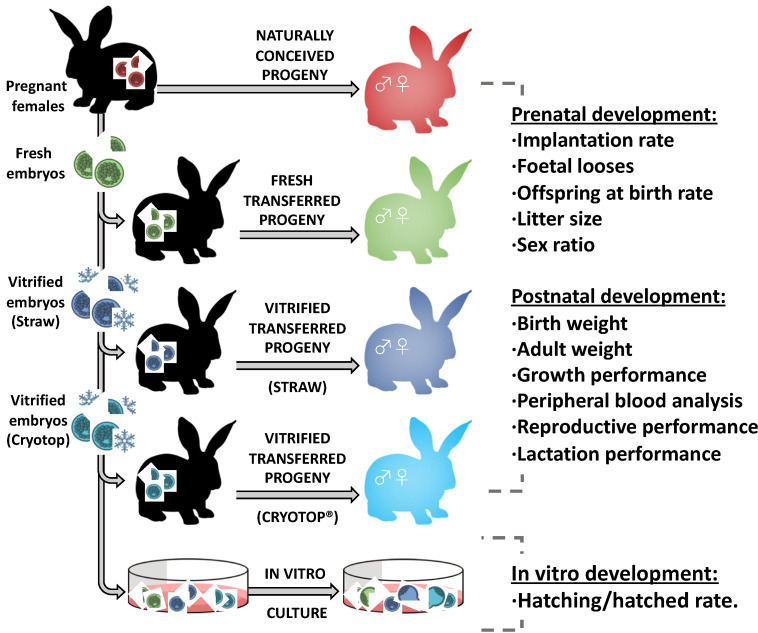
Schematic diagram of the experiment carried out to evaluate the developmental plasticity in response to embryo cryopreservation.

Figure 1 illustrates the experimental design conceived to elucidate the accumulative effects of the successive ART used in the embryo cryopreservation–transfer procedure. Accordingly, using a naturally conceived (NC) population as control group, offspring derived from fresh-transferred (FT) embryos were compared to those derived from vitrified-transferred (VT) embryos. Furthermore, VT progeny were obtained using two common clinical vitrification devices, ministraw (VTs) and Cryotop (VTc), to evaluate the influences of the cooling–warming rates provided by large vitrification volumes versus the minimum volume strategy. With this aim, a total of 22 donor females were induced to superovulate using 3 μg of corifollitropin alpha in four sessions (4–6 females per session). After 3 days, females were inseminated with semen of unrelated males with proved fertility and induced to ovulate with an intramuscular injection of 1 µg of buserelin acetate (Hoechst Marion Roussel, Madrid, Spain). Three days post-insemination, a total of 598 embryos catalogued as normal (presenting homogenous cellular mass, mucin coat, and spherical zona pellucida) were recovered *post mortem*. All embryos were pooled for later distribution in the different parts of the study, thus reducing the effect of embryo donors. Of the total, 226 and 214 embryos were subjected to vitrification/warming processes using ministraw and Cryotop as devices, respectively. After warming, only undamaged embryos (presenting homogenous cellular mass, mucin coat, and spherical zona pellucida) were kept, noting 221 (97.8% survival rate) and 211 (98.6% survival rate) embryos vitrified in ministraw and Cryotop, respectively. Then, to test the effect of each device on the embryo developmental potential, 134 ministraw-vitrified and 110 Cryotop-vitrified embryos were cultured in vitro for 48 h, evaluating their capability to reach the hatching/hatched blastocyst stage. Sixty-two fresh embryos were used as control. Of the remaining embryos, 87 ministraw-vitrified, 101 Cryotop-vitrified, and 96 fresh embryos were transferred into foster mothers (14–16 per foster mother). At birth, progenies constituted VTs, VTc, and FT groups, respectively. In addition, the NC population was established from six females inseminated the same day as the previous ones, allowing them to give birth without any ART manipulation. 

Twelve days after ovulation induction, foster females were examined by laparoscopy to determine the embryo implantation rate and to assess the foetal losses. On the day of birth, litter size per parity was annotated and compared between the experimental groups (NC, FT, VTs, VTc). After this, the four progenies were sexed and microchipped for tracking individually from birth until adulthood, comparing their growth performance. After weaning (fourth week), animals were caged collectively (eight rabbits per cage) until the ninth week. Then, animals were individually kept in separate cages (flat deck indoor cages: 75 × 50 × 40 cm). Once in adulthood (20 weeks old), the health status was assessed on the haematological and biochemical peripheral blood parameters. Male and female reproductive performance was also evaluated. Specifically, seminal traits, litter size, and lactation performance (milk yield and its composition) were assessed.

### 2.2. Embryo Vitrification

Vitrification was achieved in two steps according to previous studies [20,21,22]. Briefly, in the first step, embryos were placed for 2 min in a solution consisting of 12.5% (v/v) dimethyl sulfoxide (DMSO) and 12.5% (v/v) ethylene glycol (EG). In the second step, embryos were suspended for 1 min in a solution of 20% DMSO and 20% EG. Next, embryos suspended in vitrification medium were loaded into 0.125 mL French ministraws (IMV Technologies, L’Aigle, France) or into Cryotop (<1 µL of vitrification medium; Kitazato Corp., Shizuoka, Japan). Then, both cryodevices were plunged directly into liquid nitrogen to achieve vitrification. For warming, embryos were placed in 2 mL of 0.33 M sucrose at 25 °C to remove cryoprotectants, and washed 5 min later.

### 2.3. In Vitro Culture

A total of 244 vitrified embryos (134 in ministraw and 110 in Cryotop) and 62 fresh embryos were cultured through 3 experimental sessions during 48 h in medium TCM199 supplemented with 10% (*v*/*v*) foetal bovine serum and 1% (*v*/*v*) antibiotics (penicillin G sodium 300,000 IU/L, penicillin G procaine 700,000 IU/L, and dihydrostreptomycin sulphate 1250 mg/L; Divasa Farmavic, Barcelona, Spain). Culture conditions were 38.5 °C and 5% CO_2_ in humidified atmosphere. The in vitro development ability until hatching/hatched blastocyst stage was recorded to calculate the developmental rate (total embryos developed/total embryos cultured).

### 2.4. Embryo Transfer

Warmed or fresh embryos were laparoscopically transferred into the oviduct of asynchronous foster mothers [22], following the protocol described by Besenfelder and Brem et al. [23]. Briefly, foster mothers were anaesthetised with xylazine (5mg/kg; Rompun; Bayern AG, Leverkusen, Germany) intramuscularly and ketamine hydrochloride (35 mg/kg; Imalgene 1000; Merial S.A, Lyon, France) intravenously, and placed in Trendelenburg’s position. Then, embryos were loaded in a 17G epidural catheter, which was inserted through a 17G epidural needle into the inguinal region. Finally, while the process was monitored by single-port laparoscopy, the catheter was introduced into the oviduct through the infundibulum to release the embryos. Using this procedure, between 14 and 16 embryos were transferred in each foster mother. Both embryo vitrification and transfer processes used in this experiment were described in detail in Garcia-Dominguez et al. [22]. 

### 2.5. Prenatal Development

Twelve days after ovulation induction, foster mothers were anaesthetised as previously and examined by laparoscopy to assess the rate of transferred embryos that implanted (implantation rate). After birth, foetal loss rate and offspring rate were calculated, taking into account the relationship between litter size and the number of implanted embryos, and litter size and number of transferred embryos per female, respectively. In the NC group, the number of corpora lutea (number of oocytes released) was taken into account for estimation of available embryos. At birth, litter size and sex ratio (males/females) were recorded and compared between each progeny (NC, FT, VTs, VTc).

### 2.6. Postnatal Growth Performance and Body Weight Study

Body weights were annotated from birth to adulthood. Body weight differences between each progeny (NC, FT, VTs, VTc) were assessed at birth, 9th week (prepubertal age), and 20th week (adulthood). Growth curves were also estimated by nonlinear regression using the Gompertz equation, well suited for rabbits [24]: *y* = a exp[−b exp(−kt)]. In addition, growth rate was estimated as the average weight gain between the fourth and ninth week, a period when the rabbit growth is exponential.

### 2.7. Determination of Haematological and Biochemical Parameters of Peripheral Blood

In adulthood, 20 (10 of each sex) individual blood samples from each experimental group (NC, FT, VTs, VTc) were obtained from the central ear artery. From each animal, two blood samples were taken. The first one was dispensed into an EDTA (Ethylenediaminetetraacetic acid)-coated tube (Deltalab S.L., Barcelona, Spain), and the other into a serum-separator tube (Deltalab S.L., Barcelona, Spain). Blood count was performed from EDTA tubes, 10min after collection at the most, by using an automated veterinary haematology analyser MS 4e automatic cell counter (MeletSchloesing Laboratories, France) according to the manufacturer’s instructions. The blood parameters recorded were white blood cells, lymphocytes, monocytes, granulocytes, red blood cells, haematocrit, and haemoglobin. From the second tube, biochemical analysis of the serum glucose, cholesterol, albumin, total bilirubin, and bile acids were performed. Briefly, after blood coagulation, samples were immediately centrifuged at 3000× *g* for 10 min and serum was stored at −20 °C until analysis. Then, glucose, cholesterol, albumin, and total bilirubin levels were analysed by enzymatic colorimetric methods, whereas bile acids were estimated by photometry. All the methodologies were performed in an automatic chemistry analyser model Spin 200E (Spinreact, Girona, Spain), following the manufacturer’s instructions. All samples were processed in duplicate.

### 2.8. Male Reproductive Performance: Seminal Traits, Fertility Rate, and Induced Litter Size

Seminal traits, fertility rate, and litter size were studied. From each experimental group, 10 males began the training period with an artificial vagina at 18 weeks of age, collecting one ejaculate per week. Experimental evaluation of the males began at six months of age. One ejaculate per male was collected weekly, and ejaculates from males of the same experimental group were pooled in each session. Three 20 μL aliquots of each pool were taken. The first and second aliquots were diluted at a ratio of 1:20 with Tris-citrate-glucose extender (250 mM tris-hydroxymethylaminomethane, 83 mM citric acid, 50 mM glucose, pH 6.8–7.0, 300 mOsm/kg). The first sample was assessed for individual sperm motility and motion parameters using the Integrated Semen Analysis System version 1.0.17 (ISAS; Projectes i Serveis R + D). The system was set to record images at 25 frames/s. Then, 10 µL of the sample was placed in a 10 µm deep Makler counting chamber. Sperm motility was assessed at ×200 magnification at 37 °C using a negative phase contrast microscope. For each sample, 4 microscopic fields were analysed and a minimum of 200 sperm evaluated. The following sperm activity variables were assessed: sperm motility (%), progressive motility (%), curvilinear velocity (VCL, μm s^−1^), straight-line velocity (VSL, μm s^−1^), average path velocity (VAP, μm s^−1^), linearity coefficient (LIN; calculated as (VSL/VCL) × 100, %), straightness coefficient (STR), wobble coefficient (WOB; VSL/VAP × 100), amplitude of lateral head displacement (ALH, μm), and beat cross-frequency (BCF, Hz). The second sample was assessed for the percentage of live spermatozoa (viability, VIA) using the LIVE/DEAD sperm viability kit (Molecular Probes), which consists essentially of two DNA-binding fluorescent stains—a membrane-permeant stain, SYBR-14, and a conventional dead-cell stain, propidium iodide. The third sample was diluted at a ratio of 1:20 with 0.5% of glutaraldehyde solution in phosphate-buffered saline and observed by phase contrast at ×400 magnification to calculate the concentration, in a Thoma-Zeiss counting cell chamber, and evaluate both the percentages of intact apical ridge and abnormal sperm (on the basis of morphological abnormalities of head, neck, mid-piece, and tail).

For fertility assessment, seminal pools of each experimental group adjusted to 40 × 10^6^ spermatozoa/mL were used to perform 296 inseminations (72 NC, 77 FT, 71 VTs, and 76 VTc) in New Zealand crossbred females. Each female was inseminated with a seminal dose of 0.5 mL (20 × 10^6^ spermatozoa). At insemination time, females were injected intramuscularly with 1 μg of buserelin acetate (Hoechst Marion Roussel, Madrid, Spain) to induce ovulation. Only receptive does (red colour of vulvar lips) were inseminated, using a standard curved plastic pipette (Imporvet, Barcelona, Spain). The number of does that gave birth by number of inseminations (fertility rate) were recorded. At parturition day, the litter sizes per parity were annotated.

### 2.9. Female Reproductive Performance: Pregnancy Rate, Litter Size, and Number of Liveborns

Pregnancy rate, litter size, and number of liveborns were evaluated. For pregnancy rate assessment, seminal pools of control males were adjusted to 20 × 10^6^ spermatozoa per dose. A total of 66 receptive adult females (16 NC, 12 FT, 20 VTs, and 18 VTc) were inseminated as described above. The number of does that became pregnant by number of inseminations (pregnancy rate) was recorded. At parturition day, the litter size per parity and the number of liveborns were annotated.

### 2.10. Lactation Performance: Milk Yield, Milk Composition, and Nutritional Potential

After females had given birth, lactation performance was assessed on 41 females (10 NC, 10 FT, 11 VTs, and 10 VTc). Litters were equated to 10 kits, replacing those that died during the experiment. The milk yield was assessed on the second and third week of lactation to cover the point of maximum production. Taking advantage of the fact that rabbit pups are nursed only for about 3 min once every 24 h, milk yield was assessed using the weight-suckle-weight method [25]. First, the litters were maintained in the closed nest at 18:00. After that, the litters and the mothers were weighed before suckling at 8:00 the following day. At this time, the mothers were allowed to enter the nest and be suckled by their litters. Finally, each mother and her litter were re-weighed after suckling within 10 min. The difference in weight of each dam and its litter before and after suckling were annotated. The average of these differences was recorded as the daily milk yield of the female. 

The milk composition was analysed 2 days after the milk yield evaluation in each week. Again, the litters were kept in the closed nest at 18:00. At 8:00 on the following day, the mammary glands were shaved and disinfected with ethanol. Then, mothers were injected intramuscularly with 10 U.I. of oxytocin (Oxytocin Pituitaria Calier, Alvet Escartí S.L., Guadassuar, Valencia, Spain) to promote mammary gland contraction and milk let-down. After that, at least 15 mL of milk was collected in sterile tubes from each female by alternating manual milking between the mammary glands. Milk composition (dry matter, fat, crude protein, and lactose) was determined by mid-infrared spectroscopy using a MilkoScan FT120 (Foss Electric A/S, Hillerød, Denmark). Manual chemical methods were used to adjust the calibration lines of the equipment: desiccation (dry matter), SOXHLET (fat), and KJELDAHL (protein). Lactose content was calculated by difference with the other components. The somatic cell count (SCC) was analysed with a Fossomatic 5000 (Foss Electric A/S, Hillerød, Denmark). To test the nutritional milk value, suckling kits’ weaning weight (4 weeks of age) was recorded.

### 2.11. Statistical Analysis

Differences in binomial traits (rates of development, pregnancy, implantation, foetal losses, offspring, fertility, and sex ratio) were assessed using a probit link model with binomial error distribution, including the experimental group (NC vs. FT vs. VTs vs. VTc) and embryo transfer session (four levels) as fixed effects, and foster mother as a random effect. Meanwhile, a general linear model (GLM) was fitted for the quantitative traits (body weights, growth rate, Gompertz parameters, seminal parameters, litter size, number of liveborns, milk yield, milk composition, and milk SCC) analysis including the experimental group and embryo transfer session as a fixed effect, and foster mother as a random effect, as was done previously. For body weight analysis, sex was included as fixed effect, and litter size was used as covariate, although it remained non-significant from the ninth week of age. For milk yield and its composition, the week of extraction was used as fixed effect with two levels (second and third), and female body weight was used as the covariate for milk yield correction. A *p*-value of less than 0.05 was considered indicative of a statistically significant difference. The data are presented as least square mean ± standard error of the mean. All statistical analyses were performed with SPSS 21.0 software package (SPSS Inc., Chicago, IL, USA, 2002). 

## 3. Results

### 3.1. Effect of Embryo Vitrification on the Embryonic In Vitro Development

After 48 h of in vitro culture, the in vitro development rate of embryos vitrified in ministraw was significantly lower compared to those vitrified in Cryotop and fresh embryos (0.79 ± 0.027 vs. 0.88 ± 0.028 and 0.94 ± 0.031, for ministraw vs. Cryotop and fresh, respectively, *p* < 0.05). There were no significant differences between Cryotop and fresh groups.

### 3.2. Effect of In Vitro Embryo Manipulation during Vitrification on Implantation, Foetal Losses, Offspring Rate, and Sex Ratio

Lower implantation rate was recorded for ART progenies (FT, VTs, and VTc) compared to NC progeny (Table 1). However, compared to the FT group, a lower implantation rate was noted for VTs compared with VTc embryos. Likewise, the rate of foetal losses was higher for the VTs embryos than for all the other groups (Table 1). A higher offspring rate was recorded for the NC group than for ART animals, of which, compared to FT group, this rate was lower for VTs than for VTc embryos (Table 1). At birth, similar litter sizes were recorded between NC and FT groups, but both VT progenies showed lower values. Therefore, overall results indicated that more reduced survival rate was obtained for ART embryos. However, whereas similar trends were observed between VTc and FT embryos, those VTs showed lower prenatal survival. Female embryos could be more sensitive than males to the vitrification process because the sex ratio of VT progenies was altered in favour of males compared with the NC group. Finally, 73 NC, 71 FT, 45 VTs, and 65 VTc animals constituted the four experimental groups.

### 3.3. Postnatal Growth Performance and Body Weight

**Figure 2:**
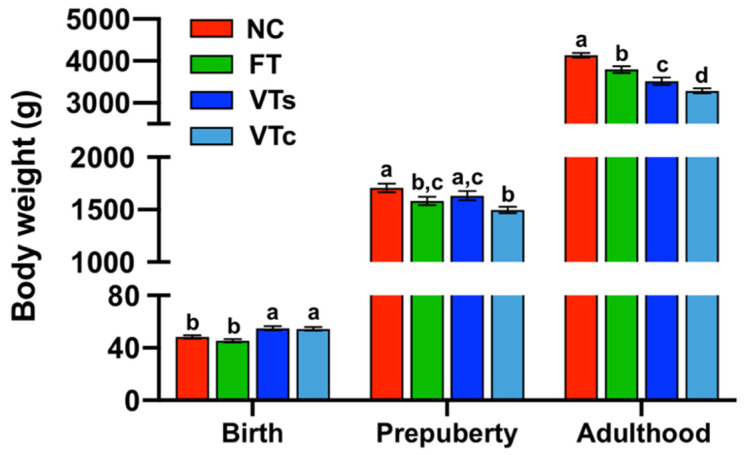
Bodyweight development: comparing differences between animals naturally conceived (NC) and those born after fresh embryo transfer (FT), vitrified embryo transfer using a ministraw (VTs), and vitrified embryo transfer using Cryotop (VTc). a,b: Bars with different superscripts differ (*p* < 0.05).

Even after using litter size as covariate, it was noted that embryo vitrification increased the birth weight, independently of the vitrification device used during the process (Figure 2; *p* < 0.05). No effects on birth weight were found in the FT group. All ART progenies showed significantly reduced body weight at adulthood compared to the NC group, with VT animals being smaller than those in FT. Adult body weight was also sensitive to the embryo vitrification methodology, of which the higher cooling–warming rates supplied by the Cryotop incurred lower body weight at adulthood, with VTs remaining heavier than VTc (Figure 2). 

**Figure 3:**
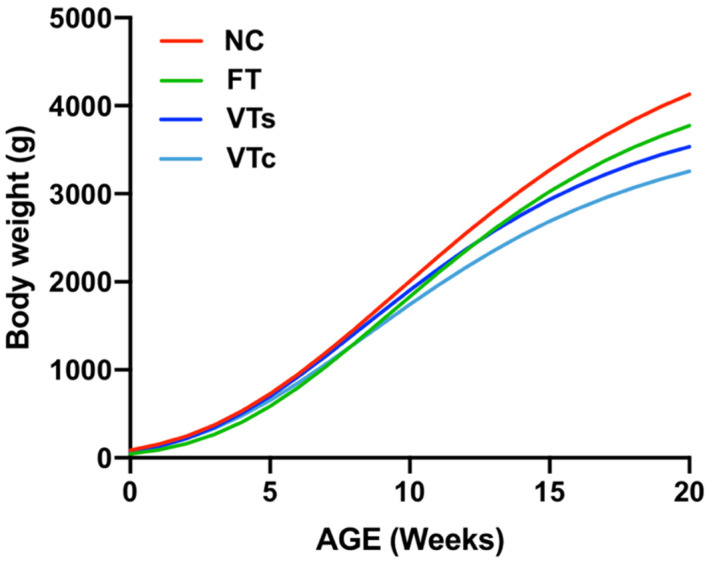
Growth curves: comparing differences between animals naturally conceived (NC) and those born after fresh embryo transfer (FT), vitrified embryo transfer using a ministraw (VTs), and vitrified embryo transfer using Cryotop (VTc).

No sexual dimorphism was observed and no interaction between treatment and sex were found. Gompertz growth curves showed a fit with a mean *r*^2^ value of 0.99 ± 0.007, describing a trend in which the growth decreased as embryonic manipulation increased (Figure 3). As estimated by the Gompertz equation (a parameter), this trend was also patent in late adulthood (4073.0 ± 98.80 g, 3792.1 ± 92.18 g, 4489.4 ± 98.64 g, and 5123.1 ± 97.74 g for VTs, VTc, FT, and NC groups, respectively; Figure 3).

In addition, estimating the growth rate in a period of exponential growth (fourth to ninth week), it was demonstrated that the postnatal growth rate was reduced in all ART groups (31.0 ± 1.4 g/day, 29.2 ± 1.01 g/day, and 32.7 ± 1.1 g/day for VTs, VTc, and FT, respectively) compared to the NC (36.2 ± 1.3 g/day) progeny (*p* < 0.05). Among ART progenies, lower growth rate was recorded for VTc animals compared to the FT group, and that of the VTs was intermediate. Therefore, differences in growth rates agreed with the differences recorded for adult body weights. The overall results indicated that both embryo transfer and vitrification processes have an impact on the offspring growth performance *per se*, with more strong effects after using high cooling–warming rates than for lower cooling–warming rates.

### 3.4. Healthy Status: Peripheral Blood Parameters

**Figure 4:**
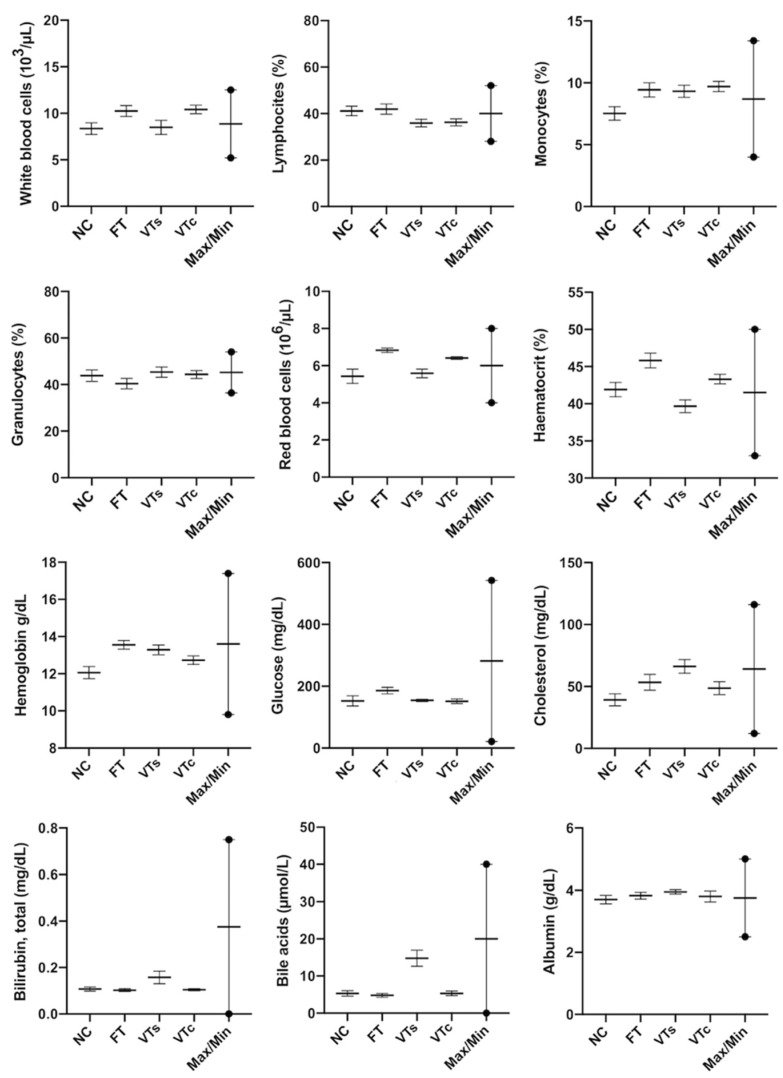
Peripheral blood analysis (haematological and biochemical): comparing differences between animals naturally conceived (NC) and those born after fresh embryo transfer (FT), vitrified embryo transfer using a ministraw (VTs), and vitrified embryo transfer using Cryotop (VTc).

From the haematological and biochemical point of view, both ART (FT, VTs, and VTc) and NC progenies seemed healthy, as peripheral blood parameters ranged between normal values in all the experimental groups (Figure 4).

### 3.5. Reproductive Performances

Regarding male reproductive performance, significant variations in the seminal concentration, progressive motility, viable sperm, straight-line velocity, linearity coefficient, wobble coefficient, and amplitude of lateral head displacement were found among the experimental progenies (Table 2). 

However, similar fertility rates and litter size recorded among the experimental groups (Table 2) indicated that, independently of its origin, sperm of sufficient quality was produced by males. Therefore, slight changes in the seminal traits were biologically irrelevant. Likewise, regarding female reproductive performance, no differences were obtained either in the pregnancy rate, litter size, or the number of liveborns (Table 3). Therefore, reproductive performance was adequate, independently of the experimental group and sex.

### 3.6. Lactation Performance

The results for lactation performance are shown in Table 3. Compared to NC animals, lower milk yield was observed in FT and VTc females. In contrast, VTs females showed a milk yield comparable to the NC females. A similar trend was observed for the milk composition analysis—whereas dry matter and fat levels were comparable between NC and VTs milk, these values were lower in the milk of FT and VTc females. Protein content was higher in VTs milk than for the other groups. On the other hand, lactose content was higher in VTc milk than for the other groups. The somatic cell count showed higher levels in VTc than in VTs and NC milk, being similar to the FT group. The nutritional potential of the milk was tested on the basis of the weaning weight of the suckling kits. Concordantly, lower weaning weights were recorded for the FT and VTc groups, compared to the NC and VTs groups (512.0 ± 10.11 g and 531.4 ± 9.58 g vs. 576.9 ± 9.46 and 561.4 ± 8.42 g, for FT and VTc vs. NC and VTs respectively; *p* < 0.05).

## 4. Discussion

Here, we describe how embryo manipulation techniques incur phenotypic changes throughout life. First, we provide long-term follow-up data of the ART cumulative effect during an embryo cryopreservation procedure. Accurately, we unravel those effects related to the cryopreservation *per se* and those associated with the embryo manipulation during the transfer procedure. Second, we report that the vitrification device effected distinct differences in the in vitro and in vivo (prenatal and postnatal) development trajectory. Third, ART animals seemed healthy due to haematological and biochemical parameters and similar reproductive performance. Therefore, we support the idea that developmental changes exhibited by ART progenies are due to an embryo developmental plasticity response.

It is well known that cells can respond to any adverse environmental condition that perturbs cellular homeostasis. Previous studies have suggested that stress during preimplantation embryo stage precipitates deviant postnatal phenotypes [10,11,12]. Overall, to be cryopreserved, embryos require exposure to an environment in which they have no intrinsic ability to survive, which exposes them to risk of a variety of types of damage or “cryoinjury” during exposure to lethal temperature [14]. Cryopreservation could thus be considered one of the most invasive ART routinely used [26]. In this article, we tested the effects of two vitrification devices on embryonic development. Our results showed similar in vitro developmental rates between fresh and Cryotop-vitrified embryos, but a lower rate for straw-vitrified embryos. Cryotop allows extremely faster cooling and warming rates in comparison with the straw devices [27,28]. Moreover, in agreement with our findings, it has been described that increasing the cooling rate improves survival rates [18,19]. Similarly, other studies based on minimum volume vitrification assays have demonstrated improved survival rates [29]. This trend was confirmed across gestation, where higher foetal loses were recorded for VTs embryos, in line with previous results [30]. A plausible explanation is that higher cryodamage induced by straw vitrification could incur in improper foetal placenta development, probably due to preferential confinement of damaged cells to the trophectoderm [31]. Remarkably, it has been described that ART impact the biological processes of placental growth, development, morphology, and function [32]. Thus, female embryos seemed more sensitive to the vitrification conditions, as skewed sex ratio towards male gender was detected at birth. This phenomenon has been well established among ART births, which has been related with abnormal inactivation in one of the two X chromosomes in females and a higher rate of irregular placentation that incurs higher mortality for female embryos [33,34,35,36]. However, in the field of embryo cryopreservation, information is limited and controversial, and changes in the sex ratio have been attributed to the grading criteria used, instead of to the cryopreservation procedure *per se* [37,38,39]. Thus, to our best knowledge, this is the first study to demonstrate in a randomised model that embryo vitrification could imbalance the offspring sex ratio in favour of males.

Further evidence for the cryopreservation impact comes from postnatal phenotypic observations. In this article, we have shown that animals born after embryo cryopreservation exhibit higher birth weight and poor growth performance independently of the tested device. Higher birth weight has been observed following embryo cryopreservation in different mammalian species, including humans [31,40,41]. Concordantly, it has been described that ART increases the risk of some foetal overgrowth syndromes, such as large offspring syndrome in bovines and Beckwith–Wiedemann syndrome in humans, both associated with epigenetic changes [42,43,44,45]. In this sense, epigenetic studies point toward differential methylation of critical genes for growth that may be responsible for the increased incidence of body weight disorders following ART [46]. In addition, epigenetic variations in ART births can remain in adulthood [26]. In this sense, in agreement with previous findings [10,11,12,47], we reported that embryo manipulation incurred a cumulative effect, leading to growth and adult body weight deviations, with more severe preimplantation stress precipitating more deviant phenotypes. These phenotypic modifications meet the concerns of ART practitioners, especially those regarding birth weight, growth trajectories, and developmental defects [10]. Today, it is well established that superovulation can also alter the epigenetic status of the resultant embryos and thereby incur long-term effects for the offspring [48]. Moreover, when different stressors exist, these can act synergistically inducing more adversarial effects. However, it is difficult to distinguish between the adverse effects caused by superovulation or by the subsequent ART, because current protocols require superovulation as an initial step. Thus, in most studies, all adverse effects are considered together as part of the ART protocol [11]. Nevertheless, ART animals are seemingly healthy, supported by the haematological and biochemical analyses of the peripheral blood, which are within the normal physiological range of variability. Furthermore, it is widely known that critical health conditions may impair the reproductive system [49]. Although potential effects of ART over reproductive traits have been described [50,51], here, reassuringly, no differences in reproductive performance were noticed between ART and NC progenies.

Throughout this study, it was demonstrated that although Cryotop has a positive effect on the in vitro and in vivo embryo survival, postnatal growth performance was severely impaired, ultimately leading to lower body weight at adulthood. A plausible explanation for this difference is that preimplantation embryos can develop a stress-dependent response when faced with different cooling–warming rates during vitrification. On the other hand, some studies suggested that embryo cryopreservation may acts as selection pressure, filtering-out ART-sensitive embryos that not sustain the stresses associated with vitrification and warming processes [52,53,54]. In this sense, and in concordance with the higher mortality exhibited by the VTs embryos, it is well stablished that straw devices provoke slower cooling–warming rates, and thus more troublesome conditions for embryo survival than Cryotop devices [18,19,27,28,29]. Therefore, straw could produce a more powerful selection pressure that ultimately selects ART-resistant embryos, originating an offspring with less deviant developmental trajectories. However, to further characterise the effect of embryo cryopreservation procedure and methodology, we analysed lactation performance through milk yield and milk composition in NC, FT, VTc, and VTs females. Herein, we find evidence that does derived from FT and VTc embryos had lower lactation performance compared with the NC group. In contrast, milk yield and composition of VTs does were not affected, remaining similar to the NC group. Increased SCC levels in VTc and FT milk suggest poorer breast health condition and milk quality in these groups [55]. Thus, overall results indicated that a more reduced lactation performance was exhibited by FT and VTc does compared to the NC group, leaving that of the VTs unaltered. To determine whether milk yield deficiency could affect body weight in suckling kits, we determined the bodyweight at four weeks of age (weaning). Consistently, the bodyweight of the suckling kits was severely impaired in FT and VTc females compared to NC and VTs females. Litter size in which rabbit does were raised before weaning did not influence their later milk yield [25]. However, reshapes in both prenatal and postnatal trajectories can influence mammary gland development in a manner that will determine milk yields during subsequent lactations [25,56]. In agreement with our findings, a recent study on in vitro embryo production showed significant changes in growth and reductions in the milk yield and fat and protein production in bovine [57]. Nevertheless, the underlying mechanisms associating embryo manipulation and milk yield or composition remains to be explored. We hypothesise that, as skewed sex ratio reflects, embryo “cryoselection” might act especially on female embryos, which could favour the inheritance of determinant alleles [58] that can be related with the proper lactation performance manifested by VTs females. As the breast milk is of fundamental importance for the short- and long-term survival of suckling new-borns, following the ART-conceived offspring could be necessary, as the effects of altered breastfeeding can be combined with a transgenerational inheritance of the ART-induced phenotypes [59,60]. Until the recent past, it was unclear whether embryo manipulation could alter health and development throughout the course of life, because for many years good fertility and the absence of malformations were the only criteria used to qualify the resulting progeny as “normal” [61]. Hence, as the long-term effects have not been considered for a long time, the information available is scarce. Although we cannot assure that embryo transfer and vitrification manipulation might cause epigenetic modifications, our results unequivocally described an example of the plasticity of early development. 

## 5. Conclusions

Together, findings from our animal model approach showed significant phenotypic changes in the adult rabbit after embryo vitrification. Hence, our study provides evidence of long-term phenotypic changes after embryo manipulation, supporting that stress during early embryo development precipitates deviant postnatal phenotypes. Moreover, to our best knowledge, this study reports the first piece of evidence demonstrating that the vitrification device used is not a trivial decision, providing valuable information about how the cooling–warming rates during vitrification can be partly responsible of postnatal phenotypic variations. In this sense, our results highlight ART as a possible trigger of the embryonic developmental plasticity manifestation in mammalian species. Although ART progenies seem healthy, further studies reaching senescence age and involving several species are needed to accumulate robust information about ART to guarantee the safety of reproductive technologies. 

## Figures and Tables

**Table 1:** Implantation rate, foetal loss rate, offspring rate, and sex ratio in offspring born after natural conception, fresh embryo transfer, vitrified embryo transfer using ministraw, and vitrified embryo transfer using Cryotop.

Traits	Naturally Conceived	Fresh-Transferred	Vitrified-Transferred	
			Ministraw	Cryotop
Embryos (*n*)	85 ^+^	96	87	101
Foster mothers (*n*)	6	6	6	7
Implantation rate	0.95 ± 0.021 ^a^	0.88 ± 0.034 ^b^	0.67 ± 0.051 ^c^	0.78 ± 0.041 ^bc^
Foetal loss rate	0.10 ± 0.033 ^b^	0.15 ± 0.039 ^b^	0.31 ± 0.061 ^a^	0.17 ± 0.042 ^b^
Offspring rate	0.86 ± 0.038 ^a^	0.74 ± 0.045 ^b^	0.52 ± 0.054 ^c^	0.65 ± 0.048 ^bc^
Litter size	12.2 ± 0.83 ^a^	11.8 ± 0.83 ^a^	7.5 ± 0.83 ^b^	9.3 ± 0.77 ^b^
Sex ratio	0.75:1 ^b^	1.08:1 ^ab^	1.33:1 ^a^	1.5:1 ^a^
Total born (*n*)	73	71	45	65

*n*: Number; ^+^ Estimated from the ovulation rate. Data are expressed as mean ± standard error of means. ^ab^ Values within a row with different superscripts differ (*p* < 0.05).

**Table 2:** Male reproductive performance: comparing differences between naturally conceived males and those born after fresh embryo transfer, vitrified embryo transfer using ministraw, and vitrified embryo transfer using Cryotop.

Traits	Naturally Conceived	Fresh-Transferred	Vitrified-Transferred	
			Ministraw	Cryotop
Semen parameters				
Pools (*n*)	13	15	12	10
CON (10^6^ spz/ml)	253.8 ± 31.71 ^ab^	317.8 ± 28.58 ^a^	217.3 ± 34.47 ^b^	248.5 ± 34.47 ^ab^
MOT (%)	88.6 ± 2.46	87.4 ± 2.37	90.3 ± 2.56	83.8 ± 2.95
PRO (%)	50.4 ± 2.87 ^ab^	43.1 ± 2.77 ^b^	53.1 ± 2.98 ^a^	42.1 ± 3.45 ^b^
VIA (%)	90.5 ± 1.60 ^a^	87.4 ± 1.55 ^ab^	84.8 ± 1.87 ^b^	89.6 ± 1.87 ^ab^
NAR (%)	95.1 ± 0.86	94.7 ± 0.80	93.1 ± 0.94	95.3 ± 1.04
ABN (%)	19.6 ± 2.01	19.1 ± 1.88	17.9 ± 2.19	17.1 ± 2.01
Motion parameters				
VCL (μm s^−1^)	98.5 ± 3.38	103.9 ± 3.11	100.3 ± 3.23	106.9 ± 3.96
VSL (μm s^−1^)	48.8 ± 2.18 ^a^	42.5 ± 2.09 ^b^	49.1 ± 2.18 ^a^	43.4 ± 2.67 ^ab^
VAP (μm s^−1^)	69.9 ± 2.25	66.1 ± 2.17	70.2 ± 2.25	67.9 ± 2.76
LIN (%)	48.5 ± 2.19 ^a^	41.2 ± 2.11 ^b^	49.1 ± 2.19 ^a^	40.6 ± 2.68 ^b^
STR (%)	69.1 ± 2.22	63.8 ± 2.04	68.1 ± 2.12	64.9 ± 2.59
WOB (%)	68.8 ± 1.67 ^ab^	64.1 ± 1.54 ^c^	69.8 ± 1.60 ^a^	64.4 ± 1.96 ^cb^
ALH (μm)	2.3 ± 0.12 ^ab^	2.3 ± 0.12 ^ab^	2.0 ± 0.12 ^b^	2.5 ± 0.15 ^a^
BCF (Hz)	9.8 ± 0.49	9.8 ± 0.47	9.9 ± 0.49	9.7 ± 0.69
Fertility rate	0.97 ± 0.019	0.94 ± 0.028	0.93 ± 0.030	0.92 ± 0.031
Litter size	12.1 ± 0.38	11.7 ± 0.40	11.9 ± 0.43	12.3 ± 0.41

*n*: number; CON: spermatic concentration; TSE: total sperm per ejaculate; spz: spermatozoa; MOT: percentage of sperm motility; PRO: percentage of progressive motility; VIA: percentage of viable sperm; NAR: percentage of normal apical ridge; ABN: percentage of abnormal forms; VCL: curvilinear velocity; VSL: straight-line velocity; VAP: average path velocity; LIN: linearity coefficient (VSL/VCL × 100); STR: straightness coefficient; WOB: wobble coefficient (VSL/VAP × 100); ALH: amplitude of lateral head displacement; BCF: beat cross-frequency. Data are expressed as least square means ± standard error of means. ^a,b^ Values within a row with different superscripts differ (*p* < 0.05).

**Table 3:** Female reproductive and lactation performance: comparing differences between naturally conceived females and those born after fresh embryo transfer, vitrified embryo transfer using ministraw, and vitrified embryo transfer using Cryotop.

Traits	Naturally Conceived	Fresh-Transferred	Vitrified-Transferred	
			Ministraw	Cryotop
Inseminated females	16	12	20	18
Reproductive performance				
Pregnant females	16	11	20	17
Litter size	10.5 ± 0.65	9.1 ± 0.69	10.2 ± 0.62	9.1 ± 0.65
Liveborn	8.5 ± 0.68	8.9 ± 0.85	8.6 ± 0.60	8.5 ± 0.66
Lactation performance				
Milk yield (g/day)	261.9 ± 12.21^a^	206.5 ± 13.44 ^b^	255.2 ± 10.98 ^a^	219.6 ± 11.26 ^b^
Dry matter (%)	36.3 ± 0.56 ^a^	33.5 ± 0.59 ^b^	36.0 ± 0.54 ^a^	33.94 ± 0.56 ^b^
Fat (%)	21.6 ± 0.51^a^	18.5 ± 0.53 ^b^	20.5 ± 0.48 ^a^	18.3 ± 0.51^b^
Protein (%)	10.9 ± 0.17 ^b^	11.0 ± 0.18 ^ab^	11.5 ± 0.17 ^a^	11.2 ± 0.18 ^ab^
Lactose (%)	2.5 ± 0.08 ^b^	2.4 ± 0.08 ^b^	2.5 ± 0.08 ^b^	2.8 ± 0.08 ^a^
Somatic cells (10^3^/mL)	371.9 ± 101.09 ^b^	557.3 ± 113.92 ^ab^	408.1 ± 98.77 ^b^	725.3 ± 101.09 ^a^

Data are expressed as least square means ± standard error of means. ^ab^ Values within a row with different superscripts differ (*p* < 0.05).

## Abbreviations

The following abbreviations are used in this manuscript:

ABN	Percentage of abnormal forms
ALH	Amplitude of lateral head displacement
ART	Assisted reproductive technologies
BCF	Beat cross-frequency
CON	Spermatic concentration
DMSO	Dimethyl sulfoxide
EDTA	Ethylenediaminetetraacetic acid
EG	Ethylene glycol
ESHRE	European Society of Human Reproduction and Embryology
FT	Fresh-transferred
GLM	General linear model
LIN	Linearity coefficient
MOT	Percentage of sperm motility
NAR	Percentage of normal apical ridge
NC	Naturally conceived
PRO	Percentage of progressive motility
SCC	Somatic cell count
Spz	Spermatozoa
STR	Straightness coefficient
TSE	Total sperm per ejaculate
VAP	Average path velocity
VCL	Curvilinear velocity
VIA	Percentage of viable sperm
VSL	Straight-line velocity
VT	Vitrified-transferred
VTc	Vitrified-transferred using Cryotop
VTs	Vitrified-transferred using ministraws
WOB	Wobble coefficient
